# Temporal trends and predictors of perfluoroalkyl substances serum levels in Swedish pregnant women in the SELMA study

**DOI:** 10.1371/journal.pone.0209255

**Published:** 2018-12-31

**Authors:** Huan Shu, Christian H. Lindh, Sverre Wikström, Carl-Gustaf Bornehag

**Affiliations:** 1 Department of Environmental Science and Analytical Chemistry, Stockholm University, Stockholm, Sweden; 2 Dept. of Health Sciences, Karlstad University, Karlstad, Sweden; 3 Division of Occupational and Environmental Medicine, Lund University, Lund, Sweden; 4 School of Medical Sciences, Örebro University, Örebro, Sweden; 5 Department of Preventive Medicine, Icahn School of Medicine at Mount Sinai, New York City, New York, United States of America; University of Pittsburgh, UNITED STATES

## Abstract

**Background:**

Perfluoroalkyl substances (PFAS) are used in numerous consumer products. They are persistent, bioaccumulating, and suspected to be endocrine disrupting chemicals (EDCs). A growing body of research has reported the association between PFAS exposure and adverse health effects. Concerns have been raised with special focus in childhood development.

**Methods:**

Perfluoroheptanoic acid (PFHpA), perfluorooctanoic acid (PFOA), perfluorononanoic acid (PFNA), perfluorodecanoic acid (PFDA), perfluoroundecanoic acid (PFUnDA), perfluorododecanoic acid (PFDoDA), perfluorohexane sulfonate (PFHxS) and perfluorooctane sulfonate (PFOS) were analyzed by LC/MS/MS in serum from 1,616 pregnant women in the Swedish SELMA study. The serum samples were collected in the first trimester (median week 10). Least square geometric means (LSGM) of PFAS were estimated for each year period for, adjusted for potential determinants including parity, fish intake in the family, and mother's age.

**Results:**

Six PFAS (PFNA, PFDA, PFUnDA, PFHxS, PFOA, and PFOS) were detected above levels of detection (LOD) in more than 99% of the SELMA women, while PFHpA, and PFDoDA were detected above LOD in 73.4% and 46.7% respectively. Parity, maternal age, maternal smoking, and fish intake during pregnancy were found to be significantly associated (p<0.05) with serum PFAS levels in the pregnant women. Finally, serum concentration of six PFAS (PFNA, PFDA, PFHxS, PFHpA, PFOA and PFOS) were significantly decreasing (range 14–31%) during the period of 30 months from 2007–2010.

**Conclusions:**

Our analysis shows that six out of eight PFAS could be identified in serum of more than 99% of SELMA subjects with a significant slightly decreasing trend for five of these compounds. Furthermore, parity, higher fish intake and mothers age are determinants for serum levels of PFAS in pregnant women.

## Introduction

Perfluoroalkyl substances (PFAS) are organic compounds used in a variety of consumer products for many decades to make everyday products more resistant to stains, grease, and water. Products containing PFAS include from non-stick frying pans, waterproof clothing and stain-resistant fabrics, food packaging (e.g., grease proof food wrapping paper or microwave popcorn bags), and cosmetics [1–3]. There are more than 3,000 PFAS on the global market, but information on total quantities and their extent of usage is unknown [4]. Less than 2% of the 3,000 PFAS were registered under the European Registration, Evaluation, Authorization and Restriction of Chemicals (REACH) legislation [4].

PFAS have not only been detected in the environment (soil and water) and in animals, but also in human blood samples throughout the world [5–10].

Over the past decade, new evidence has implicated an association between exposure for PFAS and human health and development. Johnson, Sutton [11]] included 19 datasets from nine countries in a systematic review analysis and concluded that there is “sufficient” human evidence that prenatal exposure to perfluorooctanoic acid (PFOA) reduces fetal growth. Other risks that have been reported in relation to PFAS exposure are decreased fertility [12], attention deficit disorder [13], changes to the immune system [14], and reduced humoral immune response to routine childhood immunizations [15].

Given the reported health risks, and the fact that PFAS are persistent in the environment and bioaccumulating [16], there is a growing concern of environmental PFAA exposure. Recently, a trend has started amongst global manufacturers and downstream users to replace long-chain PFAS with short-chain PFAS [17]. Long chain PFAS include acids C8 or longer and sulfonates C6 or longer. The short-chain PFAS are considered to be less toxic, less persistent, and less bio-accumulative [18] although much less is also known about human exposure.

### Aim with the study

The overall purpose for the present study was to report serum concentrations for eight PFAS in pregnant women, to identify important determinants for these PFAA levels, and finally, to analyze temporal trend in serum levels for the eight PFAS over a period of 2.5 years (2007–2010) among 1,616 pregnant women in the Swedish Environmental Longitudinal, Mother and child, Asthma and allergy (SELMA) study.

## Material and methods

### Subject and data collection

SELMA is a longitudinal pregnancy cohort study designed to investigate impacts of early life exposure to environmental factors on growth, development, and chronic diseases in children. The SELMA study recruited pregnant women in the county of Värmland, Sweden between September 2007 and March 2010. Women living in the county of Värmland, with no plans to move out of the county, and who could read Swedish were invited to participate at their first visit to the antenatal care center. During the study period 8,394 pregnant women were identified, 6,658 were eligible and 2,582 (39%) agreed to participate in SELMA.

Blood serum samples were obtained from 2,355 pregnant women (out of the 2,582 participating women) in week 3–27 of pregnancy (median week was 10 where 96.1% of the samples were collected before week 13) at their first visit at their antenatal care center in Värmland between September 2007 and March 2010, a period of about 2.5 years [19]. The current analysis was built upon 1,616 pregnant women (median week 10, 96.5% of the samples collected before week 13) for whom we had complete baseline information for the bio-statistical modelling.

Data on parity were collected from the Swedish National Birth Register. Information on fish consumption was retrieved from a food frequency questionnaire (FFQ) filled out at week 25 of the pregnancy. Women were reporting how frequently they were eating (Do not eat = 0; 1–2 servings per month = 1; 1–2 servings per week = 2; 3–4 servings per week = 3; 5–6 servings per week = 4; 1 serving per day = 5; 2 servings per day = 6; 3 or more servings per day = 7) the following seafood during their pregnancy: herring or mackerel, salmon, cod or pollock species, smoked mackerel, tuna, freshwater fish, shellfish, caviar, or “other fish”. A fish intake index was created based on the sum of all fish consumption frequencies according to the questionnaire. Data on living location was collected from questionnaires at the time of enrollment.

### Chemical analysis of PFAS

The analyses of PFHpA, PFHxS, PFOS, PFOA, perfluorononanoic acid (PFNA), perfluorodecanoic acid (PFDA), perfluoroundecanoic acid (PFUnDA) and perfluorododecanoic acid (PFDoDA) were performed, using liquid chromatography-tandem-mass-spectrometry (LC/MS/MS) at The Department of Occupational and Environmental Medicine in Lund, Sweden. A detailed description of the method is presented in Lindh, Rylander [20]. Briefly, aliquots of 100 μl serum were added to 25 μl of a water:acetonitrile (50:50) solution containing labelled internal standards. Proteins were precipitated by acetonitrile and vigorously shaking for 30 minutes. The samples were then centrifuged and the supernatant was analyzed using a LC (UFLCXR, SHIMADZU Corporation, Kyoto, Japan) connected to a hybrid triple quadrupole linear ion trap mass spectrometer (QTRAP 5500, AB Sciex, Foster City, CA, USA). The analyses of PFOA and PFOS are part of the Round Robin inter-comparison program (Professor Dr. Med. Hans Drexler, Institute and Out-patient Clinic for Occupational-, Social- and Environmental Medicine, University of Erlangen-Nuremberg, Germany) and the results were within the tolerance limits.

### Cotinine in serum as a marker for smoking

Serum samples were collected at enrollment and analysed for cotinine as a biomarker for exposure to tobacco or nicotine. A detailed description of the method was previously presented in Lindh, Rylander [20]. Briefly, aliquots of 100 μl serum were added with labelled internal standard and proteins were precipitated by acetonitrile and analysed using LC/MS/MS. Subjects were categorised as non-smokers if their cotinine levels were below 0.2 ng/mL and as active smokers if their cotinine levels were greater than 15 ng/mL. If their levels were in between, subjects were considered to be passive smokers.

### Statistical analysis

All serum levels of PFAS were log (10) transformed to improve the approximation of a normal distribution. A series of exploratory tests were implemented to identify potential predictors of PFAA serum levels. The Pearson correlation test was used to explore the unadjusted relationship between serum PFAA levels and the mother’s age, and the Spearman correlation test was used for univariate analyses of PFAA levels in relation to the fish intake index. Covariates were selected for inclusion in the multivariate analyses if they were significantly associated with four or more serum PFAS concentrations. After applied multiple regression, least square geometric means (LSGM) of PFAS concentrations by sampling year were calculated as 10^(least square means), with 95% CIs as 10^(least squares mean ± 1.96 × SE). The least square geometric means is the sampling year specific mean of PFAS concentrations after adjusting for covariates. Percent changes in PFAS concentrations by sampling year were calculated as [10^(β)– 1] × 100% with 95% CIs estimated as [10^(β ± 1.96 × SE)– 1] where β and SE are the estimated regression coefficient and standard error respectively.

The analysis was conducted using PROC GLM in SAS version 9.3 of the SAS System for Windows. Copyright 2012, SAS Institute Inc. Cary, NC, USA.

### Ethical approval

This study was approved by the Regional Ethical Review Board in Uppsala, Sweden. Individual informed written consent was obtained before each subject began participation in the study, consent from parents or guardians were also obtained for minors.

## Results

In total, 1,616 pregnant women were included in the analyses for which we had complete data for all variables. The pregnant women had a mean age of 31 years, about 66% of them lived in inner-city or sub-urban areas, and approximately 88% were non- smokers (Table 1).

**Table 1:** Description of the study population of 1,616 pregnant women in the SELMA study.

**Age (mean year, SD)**	31(4.7)
**Parity (median, range)**	2 (1–8)
**Fish intake index (mean, range)**	4 (0–12)
**Smoking status (%)**	
**None smoker**	88.2
**Passive smoker**	5.6
**Active smoker**	6.1
**Living location (%)**	
**Inner-city area / city center**	19.5
**Sub-urban areas**	46.0
**Rural areas**	31.4
**Missing**	3.0

### Serum levels of PFAS in pregnant women

The distributions of serum levels of the eight analyzed PFAS for all 2,355 and the 1,616 pregnant women in the study group are summarized in the Table 2. Five PFAS (PFNA, PFDA, PFHxS, PFOA, and PFOS) were detected in all registered SELMA subjects, while PFUnDA, PFHpA, and PFDoDA were identified in 99.6%, 73.4%, and 46.7% of the samples respectively. The geometric mean concentration for PFOS was the highest with 5.37 ng/mL (95% CI: 0.47–5.51 ng/mL), about three times higher than PFHxS (1.31 ng/mL [1.27–1.35 ng/mL]) and PFOA (1.61 ng/mL [1.56–1.65 ng/mL]).

**Table 2:** Distribution of PFAS levels (ng/mL) in prenatal serum for 2,355 pregnant women in SELMA, and in a subgroup of 1,616 pregnant women that is the study population for the current report.

Variable Name	Pregnant women in the entire SELMA study (N = 2,355) (ng/mL))						
	N (% > LOD)	LOD	Min	Median	95^th^	Max	Geometric Mean (95% Confidence Interval)
PFNA	2,355 (100)	0.01	0.06	0.52	1.30	8.70	0.53 (0.52–0.55)
PFDA	2,355 (100)	0.02	0.03	0.25	0.61	4.00	0.26 (0.25–0.26)
PFUnDA	2,345 (99.6)	0.02	<LOD	0.22	0.53	2.40	0.21 (0.20–0.21)
PFDoDA	1,064 (45.2)	0.03	<LOD	0.03	0.08	0.39	0.026 (0.025–0.027)
PFHxS	2,355 (100)	0.03	0.21	1.20	3.70	10.00	1.30 (1.28–1.34)
PFHpA	1,716 (72.9)	0.01	<LOD	0.02	0.10	0.65	0.018 (0.017–0.019)
PFOA	2,355 (100)	0.02	0.19	1.60	4.00	21.00	1.61 (1.57–1.64)
PFOS	2,355 (100)	0.06	0.17	5.30	12.00	32.00	5.24 (5.13–5.36)

### Factors associated with PFAA levels in serum of pregnant women

Women who had given birth to more children had lower levels of PFAS in serum, and this was true for all the eight studied compounds (Table 3). Mother’s age and smoking status were found to be significantly associated with four different PFAS; however, the direction of these associations seems to be different for different compounds. Higher age was associated with higher PFUnDA and PFDoDA serum levels, and lower PFHpA and PFOA serum levels. As for smoking status, PFHxS was found higher in women who were smokers, while PFDA, PFUnDA, and PFDoDA were found to be lower in smokers. Finally, we could find significant associations between serum levels of PFAS (PFDA (p<0.001), PFUnDA (p<0.001), PFDoDA (p<0.001), and PFOS (p<0.001)) and fish intake in the family.

**Table 3:** Crude associations between three potential determinants (mothers age and smoking, fish intake, and parity) and PFAS in serum (N = 1,616).

Correlation Coefficients (r)				
P-values				
PFAS^1^^)^	Age^2^^)^	Fish Intake Index^3^^)^	Parity^3^^)^	Smoking^3^^)^
**PFNA**	-0.02	**0.14**	**-0.23**	-0.05
	0.48	**< .0001**	**< .0001**	0.06
**PFDA**	0.04	**0.23**	**-0.17**	**-0.07**
	0.11	**< .0001**	**< .0001**	**0.01**
**PFDODA**	**0.12**	**0.30**	**-0.11**	**-0.10**
	**< .0001**	**< .0001**	**< .0001**	**< .0001**
**PFUNDA**	**0.16**	**0.46**	**-0.10**	**-0.12**
	**< .0001**	**< .0001**	**< .0001**	**< .0001**
**PFHXS**	-0.03	0.04	**-0.16**	**0.07**
	0.21	0.15	**< .0001**	**0.01**
**PFHPA**	-0.04	0.02	**-0.08**	-0.02
	0.09	0.35	**0.00**	0.53
**PFOA**	**-0.15**	0.00	**-0.42**	0.03
	**< .0001**	0.99	**< .0001**	0.17
**PFOS**	-0.02	**0.15**	**-0.26**	0.00
	0.32	**< .0001**	**< .0001**	0.93

1) Log10 transformed

2) Pearson correlation

3) Spearman correlation

### Time trends for serum levels of PFAS in pregnant women

Least square geometric mean levels for four PFAS (PFHxS, PFHpA, PFOA, and PFOS) were overall significantly decreased during the sampling period (Table 4) when year 2007 was used as reference period (n = 269). LSGM levels for PFHpA were decreased by almost 27% during the period (p<0.001) while levels for PFHxS, PFOS, and PFOA decreased in the range of 13–26% when comparing year 2010 with the baseline year 2007. For the other PFAS (PFNA, PFDA, PFUnDA, PFHxS), no significant time trends could be found in crude analyses for the examined period.

**Table 4:** Crude serum levels (LSGM with 95% CIs) of PFAS (ng/mL) in each of four examined time periods and percentage change in LSGM with 2007 as a reference (N = 1,616).

		2007 (n = 263)	2008 (n = 654)	2009 (n = 593)	2010 (n = 106)	p-Value (Overall Trend)
**PFNA**	%change (95%CI)	Reference	-3.5	-7.0	-8.8	0.30
** **	LSGM (95%CI)	0.57 (0.53–0.60)	0.55 (0.53–0.58)	0.53 (0.51–0.56)	0.52 (0.47–0.58)	
**PFDA**	%change (95%CI)	Reference	-3.6	-7.1	-3.7	0.20
** **	LSGM (95%CI)	0.28 (0.26–0.29)	0.27 (0.26–0.28)	0.26 (0.25–0.27)	0.27 (0.24–0.29)	
**PFUnDA**	%change (95%CI)	Reference	-4.5	0.0	13.6	0.15
** **	LSGM (95%CI)	0.22 (0.20–0.23)	0.21 (0.20–0.22)	0.22 (0.21–0.23)	0.25 (0.22–0.28)	
**PFDoDA**	%change (95%CI)	Reference	-3.7	0.0	3.7	0.34
** **	LSGM (95%CI)	0.027 (0.025–0.029)	0.026 (0.024–0.027)	0.027 (0.026–0.029)	0.028 (0.024–0.032)	
**PFHxS**	%change (95%CI)	Reference	-6.5	-27.5***	-13.1*	**<0.0001**
** **	LSGM (95%CI)	1.53 (1.43–1.64)	1.43 (1.36–1.49)	1.11 (1.06–1.16)	1.33 (1.19–1.49)	
**PFHpA**	%change (95%CI)	Reference	-13.6*	-22.7***	-27.3**	**<0.0001**
** **	LSGM (95%CI)	0.022 (0.020–0.025)	0.019 (0.017–0.020)	0.017 (0.015–0.018)	0.016 (0.013–0.020)	
**PFOA**	%change (95%CI)	Reference	-7.3	**-13.0** ***	-15.8**	**<0.01**
** **	LSGM (95%CI)	1.77 (1.65–1.89)	1.64 (1.57–1.71)	1.54 (1.48–1.61)	1.49 (1.34–1.66)	
**PFOS**	%change (95%CI)	Reference	-6.5	-24.2***	-25.8***	**<0.0001**
** **	LSGM (95%CI)	6.2 (5.9–6.6)	5.8 (5.6–6.1)	4.7 (4.5–4.9)	4.7 (4.5–5.1)	

*p<0.05

** p<0.01

*** p<0.001

**Fig 1:**
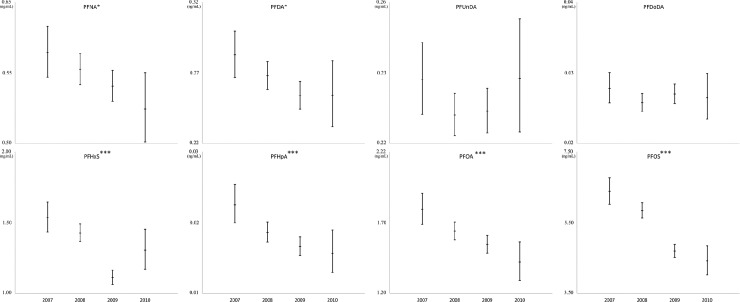
Time trends for serum levels (LSGM with 95% CIs) of PFAS (y-axis) during four periods from 2007 to 2010 (N = 1,616). The model adjusted for parity, mother age, and fish intake in the family. *p<0.05 ** p<0.01*** p<0.001.

After adjustments for parity, mother’s age, smoking status, and fish intake, least square geometric mean levels for six PFAS (PFNA, PFDA, PFHxS, PFHpA, PFOA, and PFOS) were overall significantly decreased during the sampling period (Table 5 and Fig 1). Clear and significant downward trends were found for PFOS and PFHpA around 30%, whereas PFOA decreased around 20% during the examined period of 2.5 about years (2007–2010). When comparing 2008, 2009, and 2010 levels with the baseline year 2007, LSGM of PFNA and PFHxS decreased during the last two sampling years (2009 and 2010) with 8 to 28%. A similar decreasing LSGM trend were not found for PFUnDA and PFUDoDA.

**Table 5:** Adjusted serum levels (LSGM with 95% CIs) of PFAS (ng/mL) in each of four examined time periods and percentage change in LSGM with 2007 as a reference (N = 1,616), adjusted for parity, mother age, smoking, and fish intakes.

		2007 (n = 263)	2008 (n = 654)	2009 (n = 593)	2010 (n = 106)	p-Value (overall)
**PFNA**	%change	Reference	-4.1	-8.3*	-13.8**	**0.03**
** **	LSGM (95%CI)	0.58 (0.54–0.62)	0.55 (0.53–0.58)	0.53 (0.51–0.55)	0.50 (0.45–0.55)	
**PFDA**	%change	Reference	-3.6	-10.7**	-10.7	**0.02**
** **	LSGM (95%CI)	0.28 (0.27–0.30)	0.27 (0.26–0.28)	0.25 (0.24–0.26)	0.25 (0.23–0.28)	
**PFUnDA**	%change	Reference	-6.6	-5.9	0.2	0.26
** **	LSGM (95%CI)	0.23 (0.21–0.24)	0.21 (0.20–0.22)	0.21 (0.20–0.22)	0.23 (0.20–0.25)	
**PFDoDA**	%change	Reference	-7.3	-2.9	-4.7	0.36
** **	LSGM (95%CI)	0.028 (0.026–0.030)	0.026 (0.025–0.027)	0.027 (0.026–0.028)	0.026 (0.023–0.030)	
**PFHxS**	%change	Reference	-7.1	-27.5***	-15.0**	**<0.0001**
** **	LSGM (95%CI)	1.54 (1.43–1.64)	1.43 (1.37–1.49)	1.11 (1.06–1.16)	1.30 (1.17–1.45)	
**PFHpA**	%change	Reference	-13.6**	-22.7***	-27.3**	**0.0002**
** **	LSGM (95%CI)	0.023 (0.020–0.025)	0.019 (0.017–0.020)	0.017 (0.015–0.018)	0.016 (0.013–0.019)	
**PFOA**	%change	Reference	-8.6**	-13.8***	-20.7***	**<0.0001**
** **	LSGM (95%CI)	1.79 (1.69–1.91)	1.64 (1.58–1.70)	1.55 (1.48–1.61)	1.42 (1.29–1.56)	
**PFOS**	%change	Reference	-8.5**	-26.4***	-30.8***	**<0.0001**
** **	LSGM (95%CI)	6.4 (6.0–6.8)	5.8 (5.6–6.1)	4.7 (4.5–4.9)	4.4 (4.0–4.8)	

*p<0.05

** p<0.01

*** p<0.001

## Discussion

Six out of eight examined PFAS (PFNA, PFDA, PFUnDA, PFHxS, PFOA, and PFOS) were detected in more than 99% of the SELMA subjects’ serum during the period 2007 to 2010. In a recent paper, the arithmetic mean serum levels in Swedish women from the period 1978 to 2001 were reported to be 17 ng/mL for PFOS, 2.3 ng/mL for PFOA and 0.31 ng/mL for PFNA [21]. In the present study, we found GM levels of 5.37 ng/mL for PFOS, 1.61 ng/mL for PFOA, and 0.55 ng/mL for PFNA for the period 2007–2010, indicating that the levels of PFOS and PFOA have decreased during the last decade, while PFNA levels might have increased.

In 2000, the 3M Company (the primary international manufacturer of PFOS) began a voluntary phase out of PFOS completed by 2003. A ban on most use of PFOS in the US was introduced in 2000, and in the European Union a ban came in 2008. In 2006, a voluntary agreement was reached between US regulators and eight companies to reduce emissions of PFOA by 95% by 2010. Our data indicates that these actions on PFOS and PFOA have reduced human levels for these compounds. Our data also indicate that the serum levels of PFNA, PFDA, and PFUnA have now also started to decline in Sweden. Even though our data of PFNA is showing a decreasing trend during the sampling years, several other studies have reported an increasing trend [17, 22].

We found that higher parity was associated with lower serum levels of all eight examined PFAS. Such findings are similar with what others have reported. Fei, McLaughlin [23] reported that PFOS and PFOA levels in maternal plasma were significantly negatively associated with parity. Ode, Rylander [21] reported multiparous women had significantly lower PFOA concentrations than primiparous women, and Washino, Saijo [24] reported serum concentrations of PFOS and PFOA decreased with increasing parity. Because PFAS are persistent compounds, presumably with increasing age, the concentration of PAAs in the body should be higher due to bioaccumulation. We found such a positive association to the women’s age for PFUnDA and PFDoDA, while we found a negative association PFOA. Elimination through breast milk is believed to be involved in this association, therefore postnatal exposure by breastfeeding should be a concern [25, 26].

We further found that a diet with a higher fish intake was associated with serum levels of PFNA, PFDA, PFUnDA, PFDoDA, and PFOS. Fish consumption has been identified as a source for human PFOS exposure in several other studies [2, 27–29]. In Sweden, Berger, Glynn [30] analyzed muscle tissue from edible fish species caught in Lake Vättern and in the Baltic Sea, PFOS intake was strongly dependent on individual fish consumption.

In SELMA, women who smoke during pregnancy had lower PFAS (PFDA, PFUnDA, and PFDoDA) and higher PFHxS serum level in the smokers than the non-smokers. In agreement with our study, Cho, Lam [31] reported that PFHxS was higher in the Korean population of smokers. Bjerregaard-Olesen, Bach [32] indicated that Danish women who smoked during pregnancy had lower levels of all the PFAS, including PFDA, PFUnDA, and PFHxS, compared to the women who did not smoke during pregnancy. The same correlations were not found in other studies [21, 33].

## Conclusions

Six out of eight examined PFAS (PFNA, PFDA, PFHxS, PFUnDA, PFOA, and PFOS) were detected in more than 99% of 1,616 pregnant women in Sweden during the period 2007 to 2010. Women who had given birth to more children had lower levels of PFAS in serum for all the eight studied compounds. PFUnDA and PFDoDA serum levels were found to be higher in older mother, but lower in women who were smokers. Finally, we could find significant associations between fish intake in the family and serum levels of PFDA, PFUnDA, PFDoDA, and PFOS.

## Abbreviations

LSGM: Least square geometric means
PFAS: Perfluoroalkyl substances
PFDA: Perfluorodecanoic acid
PFDoDA: Perfluorododecanoic acid
Pham: Perfluoroheptanoic acid
PFHxS: Perfluorohexane sulfonate
PFNA: Perfluorononanoic acid
PFOA: Perfluorooctanoic acid
PFOS: Perfluorooctane sulfonate
PFUnDA: Perfluoroundecanoic acid
SELMA Study: **S**wedish **E**nvironmental **L**ongitudinal, **M**other and child, **A**sthma and allergy study
